# First identification of coexistence of bla_NDM-1_ and bla_CMY-42_ among Escherichia coli ST167 clinical isolates

**DOI:** 10.1186/1471-2180-13-282

**Published:** 2013-12-05

**Authors:** Xueqing Zhang, Danping Lou, Yuanyuan Xu, Yongpeng Shang, Dan Li, Xiaoying Huang, Yuping Li, Longhua Hu, Liangxing Wang, Fangyou Yu

**Affiliations:** 1 Department of Laboratory Medicine, The First Affiliated Hospital of Wenzhou Medical University, 2 Fuxue lane, Wenzhou 325000, China; 2 Department of Respiratory Medicine, The First Affiliated Hospital of Wenzhou Medical University, 2 Fuxue lane, Wenzhou 325000, China; 3 Department of Laboratory Medicine, The Second Affiliated Hospital of Nanchang University, 1 Mingde Road, Nanchang 336000, China

## Abstract

**Background:**

Emergence of multidrug resistance in *Enterobacteriaceae* limits the selection of antimicrobials for treatment of infectious diseases. Identification of NDM-1 makes more difficulty in treating multidrug-resistant *Enterobacteriaceae* infections. Carbapenem-resistant *Escherichia coli* clinical isolates from a tertiary hospital in Wenzhou, east China, were investigated for NDM-1 production.

**Results:**

The two tested isolates were negative for modified Hodge test, but positive for a double-disc synergy test used for detecting metallo-β-lactamase production. *E. coli* WZ33 and WZ51 exhibited discrepant-level resistance to most clinically frequent used antimicrobials, but still susceptible to trimethoprim/sulfamethoxazole, amikacin, fosfomycin, tigecycline and polymyxin B. *E. coli* WZ33 and WZ51 were positive for *bla*_NDM-1_ determined by PCR and DNA sequencing. Other than *bla*_NDM-1_, *E. coli* WZ33 also harbored *bla*_CTX-M-14_ and *bla*_CMY-42_, while *E. coli* WZ51 simultaneously harbored *bla*_SHV-12,_*bla*_CTX-M-14_ and *bla*_CMY-42_. Carbapenem resistance for *E. coli* WZ51 and WZ33 could not be transferred to *E. coli* recipients through conjugation, but could be transferred to *E. coli* recipients by chemical transformation. The EcoR1-digested DNA pattern of plasmids from the transformant of *E. coli* WZ51 was different from that of *E. coli* WZ51. MLST showed that *E. coli* WZ33 and WZ51 belonged to an animal-associated clone (ST167).

**Conclusion:**

The present study is the first report of *bla*_NDM-1_ carriage in *E. coli* ST167 isolates and coexistence of *bla*_NDM-1_ and *bla*_CMY-42_ in same isolate. Systemic surveillance should focus on the dissemination of *bla*_NDM-1_ among Enterobacteriaceae, especially *E. coli* ST167 clone associated with animal infection.

## Background

*Enterobacteriaceae*, particularly *Escherichia coli* and *Klebsiella pneumioniae*, are common pathogens causing nosocomial infections. Multidrug resistance (MDR) for *Enterobacteriaceae* has been increasing rapidly and limits the selection of antimicrobials for empiric treatment of infections caused by these organisms, which is becoming a threat to public health [1]. Carbapenems are the choice for the treatment of infections caused by MDR *Enterobacteriaceae,* especially extended-spectrum β lactamase (ESBL)- and/or plasmid-mediated AmpC (pAmpC)-producing organisms. However, worldwide emergence of carbapenem resistance challenges the treatment of severe infections using carbapenems [1]. Carbapenemases, particularly the Ambler class A K. pneumoniae carbapenemases (KPCs) and the Ambler class B metallo-β-lactamases (MBLs), were mainly associated with carbapenem resistance among *Enterobacteriaceae*[2]. The genes encoding these carbapenemases are commonly located on large mobile plasmids with other determinants conferring resistance to other class antimicrobials, which facilitates the transfer of MDR to other organisms [1]. KPC-2 is found to be predominant carbapenemase among *Enterobacteriaceae*[2]. IMP- and VIM-type MBLs were another frequently described carbapenemases in *Enterobacteriaceae* worldwide [3]. Importantly, in 2009, a novel MBL, named New Delhi metallo-β-lactamase-1 (NDM-1), was identified in a *K. pneumoniae* isolate from a patient with urinary tract infection who had returned to Sweden from India [4]. Since the first report of NDM-1, this important carbapenemase was found among many species of Gram-negative rods from several countries [5-10], which has been becoming as a major public health threat and represents a new challenge for the treatment of infectious diseases. In China, NDM-1 was first identified in 4 clonally unrelated *Actinetobacter baumannii* isolates [11]. Subsequently, it was found among non-baumannii *Acinetobacter spp.* from China [12-14]. Although NDM-1 was initially found among *Enterobacteriaceae*, it has not be described in these organisms until recently in China [15,16].

Our previous study described two clonally unrelated *K. pneumoniae* isolates harboring *bla*_NDM-1_ from two teaching hospitals in Nanchang, central China [16]. In the present study, we identified *bla*_NDM-1_ among two clonally related *E. coli* isolates belonging to ST167 from one tertiary hospital in Wenzhou, east China, among which *bla*_NDM-1_ was found to coexist with *bla*_CTX-M-14_ and *bla*_CMY-42_.

## Methods

### Isolation and identification of bacterial strains

Clinical samples for microbiological examination were carefully collected by sterile containers without contamination with commensals or from external sources. Before the collection of sputum samples, patients should wash oral cavity three times using sterile physiological saline. When collecting urine samples, the meatus urinarius must be washed thoroughly for avoiding the contamination by colonizing bacteria and mid-stream urine was collected in sterile container for bacterial culture. After collection, clinical samples were transported immediately to clinical laboratory for microbiological examination. Sputum samples observed <10 squamous cells and >25 white blood cells per visual field under microscope with 100 times magnification were qualified for bacterial culture. The qualified samples were inoculated on blood agar plate for the isolation of bacteria in accordance with routine procedure. The bacterial isolates from sputum samples with amount of >10^7^ CFU/ml and from urine samples with amount of >10^5^ CFU/ml by quantitative culture were considered to be responsible for infection. Identification of bacterial isolates was performed using Vitek-2 automated microbiology analyzer (bioMe’rieux, Marcy l’Etoile, France) according to the manufacturer’s instructions. *Staphylococcus aureus* ATCC25923 and *E. coli* ATCC 25922 were used as quality control strains for bacterial identification. Written informed consent for participation in the study was obtained from participants. The Ethics Committee of the first Affiliated Hospital of Wenzhou Medical University exempted this study from review because the present study focused on bacteria.

### Antimicrobial susceptibility testing

Antimicrobial susceptibility test was performed initially using Gram-negative susceptibility (GNS) cards on the Vitek system (bioMe’rieux, Marcy l’Etoile, France). The E-test method was used for further determination of minimum inhibitory concentrations (MICs) of clinically important antimicrobial agents for clinical isolates and their transformants, in accordance with manufacturer’s instructions. Antimicrobials evaluated included ampicillin, amikacin, gentamicin, levofloxacin, piperacillin, piperacillin/tazobactam, cefotaxime, ceftazidime, cefepime, aztreonam, cefoxitin, imipenem, meropenem, ertapenem, tigecycline, polymyxin B, fosfomycin and trimethoprim/sulfamethoxazole. Results of susceptibility testing were interpreted in accordance with the criteria recommended by Clinical and Laboratory Standards Institute (CLSI) [17]. *S. aureus* ATCC25923 and *E. coli* ATCC 25922 were used as quality control strains for susceptibility testing.

### Detection of β lactamase production

The modified Hodge test (MHT) was performed on a Mueller-Hinton agar plate with ertapenem as substrate and *E. coli* ATCC 25922 as the indicator organism for detection of carbapenemases as described previously [17]. A double-disc synergy test was designed for detecting MBLs as described previously [18]. Briefly, imipenem and combined imipenem with EDTA (750 μg) disks were placed on the agar plates with the tested isolates. After over-night incubation at 35°C, if inhibition zone diameter of the imipenem with EDTA disk increases ≥6 mm relative to imipenem disk, the test is considered positive. ESBL production was determined by the CLSI-recommended confirmatory double disk combination test [17]. Isolates were tested for AmpC activity by a three-dimensional extract method as described previously [19].

### Detection of antimicrobial resistance determinants

Potential antimicrobial resistance determinants including carbapenemase genes, ESBL genes, plasmid-mediated AmpC genes and plasmid-mediated quinolone resistance determinants were investigated using the polymerase chain reaction (PCR) and nucleotide sequencing, employing previously published primers [20-24]. Plasmid Midi kits (Qiagen, Hilden, Germany) were used to extract plasmid DNA from donors and transformants according to the manufacturer’s instructions. Plasmid DNA of transformants was digested by EcoR1 according to manufacturer’s instructions. 10 μl of each digestion mixture was subjected to electrophoresis on 1.0% agarose gels, stained with ethidium bromide, and photographed under UV light.

#### Transferability of plasmids with carbapenem resistance

In order to determine whether carbapenem resistance was transferable in *E. coli* isolates, a conjugation experiment was performed using *E. coli* J53 (azide resistance) as the recipient as previously described [25]. Transconjugants were selected on tryptic soy agar plates containing sodium azide (100 μg/ml) for counterselection, and imipenem (0.5 μg/ml) for plasmid-mediated carbapenem resistance selection. Standard heat-shock transformation of chemically competent bacteria was applied to transfer carbapenem resistance. Briefly, 5 μl of DNA (25 ng) was mixed into 50 μl of competent cells (*E. coli* DH5α) in a microcentrifuge tube. After placing competent cells and DNA mixture on ice for 30 min, 2/3 of the tube was placed into a 42°C water bath for 45 seconds. The tube was put back on ice for 2 min. 500 μl of Luria-Bertani media without antibiotic was added into the tube and the mixture grew in 37°C shaking incubator for 45 min. All of the transformation were plated onto Luria-Bertani agar plates containing imipenem (0.5 μg/ml) and incubated at 37°C overnight.

### Multi-locus sequence typing (MLST)

MLST were performed on E. coli isolates positive for *bla*_NDM-1_ using amplification of internal fragments of the seven housekeeping genes of *E. coli* according to the *E. coli* MLST website (http://mlst.ucc.ie/mlst/dbs/Ecoli).

## Results and discussion

### Bacterial isolation and patients’ information

In August, 2012, *E. coli* WZ33 with carbapenem resistance was isolated from urine of a 43-year–old female patient with infectious symptoms at the First Affiliated Hospital of Wenzhou Medical University (FAHWMU) in Wenzhou, central China. FAHWMU is the largest comprehensive hospital with 3000 beds in Wenzhou. On July 11, 2012, the patient diagnosed with acute myelitis was admitted to FAHWMU. After hospitalization, she was subjected to urethral catheterization. Subsequently, she appeared infectious symptoms on July 20, with the highest body temperature of 39.5°C. Urine and blood of the patient were collected on July 20 and 21 for microbiological culture. A carbapenem-susceptible *E. coli* isolate with only resistance to ampicillin, gentamycin, tobramycin and trimethoprim/sulfamethoxazole was isolated from urine sample, while another carbapenem-susceptible *E. coli* isolate with same resistance profiling as that of the isolate from urine sample was isolated from blood sample. The patient’s symptoms improved following the treatment with cefuroxime and ceftazidime via intravenous drip. On August 6, urine sample was collected for microbiological culture again. Surprisingly, a carbapenem-resistant E. coli isolate with pure growth, named *E. coli*WZ33, was isolated from urine sample. After subjected to be treated with antimicrobials for 5 days, the symptoms of the patient disappeared and she was discharged from the hospital. The other carbapenem-resistant isolate *E. coli*WZ51 was isolated from the sputum of a 66-year-old male patients with pulmonary infection at FAHWMU. Before admitted to FAHWMU, the patient was hospitalized at another comprehensive hospital away from FAHWMU about 30 kilometers for anti-infection therapy using levofloxacin. After hospitalization at FAHWMU on March 19, the patient was subjected to treatment of pulmonary infection using ceftazidime via intravenous drip. On March 20, sputum sample was collected for bacterial culture and carbapenem-resistant isolate, *E. coli*WZ51, was identified later. After subjected to be treated with ceftazidime for 4 days, the symptoms of the patient disappeared.

### Antimicrobial resistance determinants

As both *E. coli* WZ33 and WZ51 were resistant to third-generation cephalosporin and carbapenems, MHT was performed to determine the production of carbapenemases. Unexpectedly, both tested isolates were MHT negative. For further investigation on carbapenemase production, a double-disc synergy test was used for detecting the MBL production. As expected, both tested isolates were found to produce MBLs. The genes encoding carbapenemases, including *bla*_VIM_, *bla*_IMP_, *bla*_SPM-1_, *bla*_GIM-1_, *bla*_SIM-1_ and *bla*_NDM-1_, were further investigated by PCR and DNA sequencing. Two carbapenem-resistant isolates with carbapenemase production, *E. coli* WZ33 and WZ51, were positive for *bla*_NDM-1_. The MHT has an excellent sensitivity for detecting enterobacterial isolates producing KPC- and OXA-48-type carbapenemases, but has low sensitivity for the detection of NDM-1 producers [26]. Previous study reported that negative or weakly positive MHT results were observed for 11 of 15 NDM-1-producing strains [27]. Two NDM-1-producing *K. pneumonia* clinical isolates reported by our previous study were also MHT negative [16]. In the present study, two NDM-1-producing *E. coli* isolates were also negative for MHT. The false negative results of MHT in NDM-1-producing isolates may be caused by the production of some substances that can inhibit growth of *E. coli* ATCC25922 and low production of NDM-1 [27]. False positive results of carbapenemase production by the MHT among isolates with resistance or reduced susceptibility to carbapenem result from low-level carbapenem hydrolysis by CTX-M type ESBLs and ESBL production coupled with porin loss [28,29]. These data mentioned above indicated that the detection of carbapenemases by the MHT was challenged, especially the detection of NDM-1. NDM-1 was mainly found in Enterobacteriaceae in south Asia, Europe and America [5,6,30]. In contrast, it was initially and mainly described in *Actinetobacter spp.* clinical isolates in China [11-14]*, *even emergence of dissemination of NDM-1-producing *A. pittii* (27 isolates) in an intensive care unit [31]. Recently, a higher isolation of NDM-1-producing *A. baumannii* from the sewage of the hospitals in Beijing*,* the capital of China, was described, indicating that the hospital sewage may be one of the diffusion reservoirs of NDM-1 producing bacteria [32]. However, one screening effort revealed no *bla*_NDM-1_ expression among 3439 *E. coli* and 2840 *K. pneumoniae* isolates from 57 hospitals representing 18 provinces in China [11]. Recently, *bla*_NDM-1_ began to emerge in Enterobacteriaceae from China [15,16,33]. Two clonally unrelated *K. pneumoniae* isolates from two teaching hospitals in Nanchang, central China, were found to harbor *bla*_NDM-1_[16]. Coexistence of *bla*_NDM-1_ and *bla*_IMP-26_ was identified among a carbapenem-resistant *Enterobacter cloacae* clinical isolate from southwest China [33]. Sporadic emergence of *bla*_NDM-1_ in *E. coli* clinical isolates in the present study further corroborates the evidence that *bla*_NDM-1_ carriage extends beyond *Actinetobacter spp* into Enterobacteriaceae in China*.* Another study from China also found that a *E. coli* clinical isolate isolated from the ulcer secretion of patient with diabetes-related foot complications harbored *bla*_NDM-1_[15]. International travelers to the Indian subcontinent, are prone to acquire the infections caused by NDM-1-producing organisms [4,5]. However, the two patients harboring NDM-1-producing *E. coli* had never traveled to outside China.

### Antimicrobial susceptibility profiling

The results of antimicrobial susceptibility of *E. coli* WZ33 and WZ51 are listed in Table 1. Both tested isolates were multi-resistant to clinically frequently used antimicrobials, including ampicillin, piperacillin, piperacillin/tazobatam, cefotaxime, ceftazidime, cefepime, cefoxitin, aztreonam, imipenem, meropenem, ertapenem and gentamicin, levofloxacin, but susceptible to trimethoprim/sulfamethoxazole, amikacin, fosfomycin, tigecycline and polymyxin B. Most of NDM-1-producing isolates were highly resistant to clinically available antibiotics except to tigecycline and colistin [4].

**Table 1:** MIC values of antimicrobials for *E.coli* isolates carrying blaNDM-1 and their transformants

**Antimicrobials**	**MIC values (μg/ml)**				
	***E. coli *** **WZ33**	^***a***^ ***E.coli *** **DH5α (WZ33)**	***E.coli *** **WZ51**	^***a***^ ***E. coli *** **DH5α (WZ51)**	***E. coli *** **DH5α**
ampicillin	>256	>256	>256	>256	1.5
piperacillin/tazobatam	>256	16	>256	256	0.75
piperacillin	>256	16	256	>256	0.038
ceftazidime	>256	>256	>256	>256	0.094
cefotaxime	>256	64	>256	192	0.047
cefepime	>256	16	>256	4	0.047
aztreonam	32	0. 023	>256	12	0.023
cefoxitin	>256	>256	>256	>256	0.75
imipenem	8	6	24	12	0.094
meropenem	>32	6	>32	3	0.016
ertapenem	>32	24	>32	4	0.008
amikacin	1.5	0.75	2	0.50	0.50
gentamicin	24	0.38	16	0.125	0.125
levofloxacin	24	0.047	≥32	0.016	0.023
trimethoprim/sulfamethoxazole	0.75	0.008	>32	0.008	0.008
polymyxin B	1.5	0.38	1.5	0.38	0.38
tigecycline	0.19	0.5	1	0.19	0.19
Fosfomycin	0.5	0.25	2	0.94	0.94

a, transformant.

Co-production of carbapenemases with other β-lactamases including ESBLs and pAmpCs results in resistance to nearly all clinically available β-lactams. As both *E. coli* WZ33 and WZ51 were highly resistant to all tested β-lactams, other β-lactamases other than NDM-1 were investigated. Although a ESBL gene *bla*_CTX-M-14_ was identified in *E. coli* WZ33 and two ESBL genes, *bla*_CTX-M-14_ and *bla*_SHV-12_, were found in *E. coli* WZ51, ESBL production was not detected in these two isolates, determined by CLSI-recommended double-disk test. As carbapenemases and AmpCs are not inhibited by clavulanic acid, co-production of ESBLs, AmpCs and carbapenemases can mask determination of ESBLs using the CLSI-recommended double-disk test [17]. Both *E. coli* WZ33 and WZ51 were highly resistant to cefoxitin (MICs ≥ 256), which was indicative of AmpC production. As expected, two tested isolates were found to harbor pAmpC gene *bla*_CMY-42_ in accordance with phenotypic results determined by three-dimension test. *bla*_CMY-42_ was first identified in a *E. coli* isolate [34]. The present study is the second report of *bla*_CMY-42_. However, it is the first report of the coexistence of *bla*_CMY-42_ and *bla*_NDM-1_.

### Transferability of resistance plasmids carrying bla_NDM-1_

*bla*_NDM-1_ was found to be located on the plasmids with different size and genetically diverse background and disseminated among different species of organisms by the transfer of resistance plasmids [1,5]. The plasmids conferring carbapenem resistance for *E. coli* WZ33 and WZ51 were not successfully self-transferred into the recipient *E. coli* J53 using filter mating conjugation by repeat attempts. But the plasmids conferring carbapenem resistance for both ***E. coli*** WZ33 and WZ51 could be transferred into the recipient (*E. coli* DH5α) using chemical transformation. WZ33 contained 2 plasmids (approximately 65- and 3-kb). WZ51 contained 3 plasmids with sizes of approximately 65-, 7- and 3-kb). The transformants each contained a single *bla*_NDM-1_-bearing plasmid with size of approximately 65 kb.

The transformant from *E. coli* WZ51 was positive for *bla*_NDM-1_ and *bla*_SHV-12_, while the transformant from WZ33 carrying only the NDM gene was susceptible to aztreonam, which is characteristic of MBLs.

**Figure 1:**
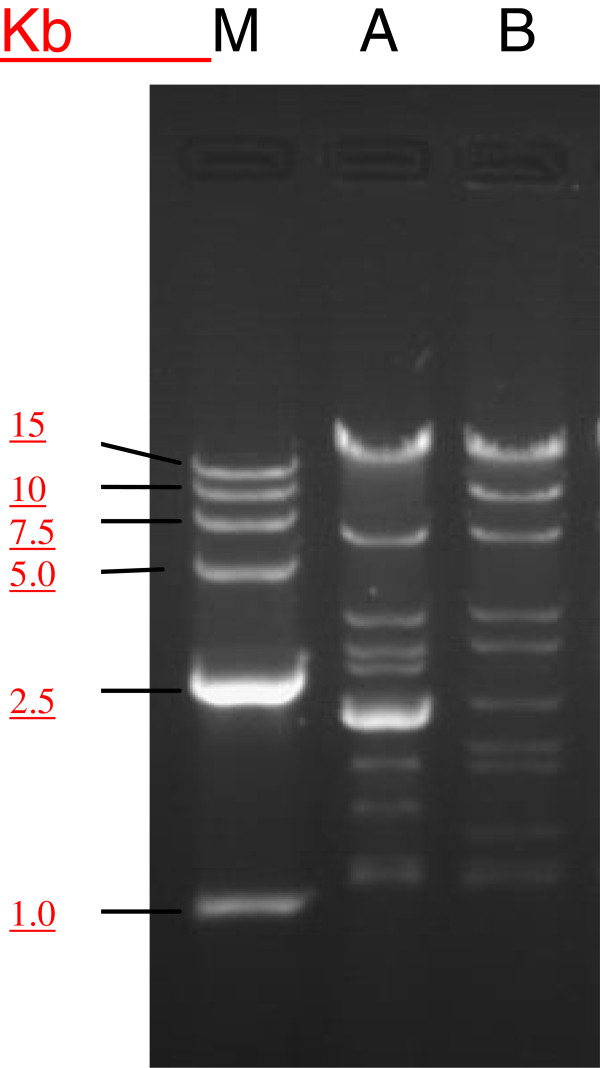
***EcoR1-digested DNA patterns of plasmids from the transformants of E. coli *WZ33 and WZ51*. *M,** the DNA ladder; **A**, Digest of plasmid from the transformant of *E.coli* WZ33; **B,** Digest of plasmid from the transformant of *E.coli* WZ51.

The transformants of two tested isolates showed increased MICs for antimicrobials relative to the recipients (*E. coli* DH5α) (Table 1). The plasmids with carbapenem resistance in *E. coli* WZ33 and WZ51 exhibited discrepant MICs for antimicrobials, indicating that the two plasmids may be different kind of plasmids. As expected, the EcoR1- digested DNA pattern of plasmids from the transformant of *E. coli* WZ33 was different from that for *E. coli* WZ51 (Figure 1). Connections between sequence types and plasmid replicons are potentially important. *K. pneumoniae* ST14 clone was found to be important for the dissemination of *bla*_NDM-1_[35].

### Genotype of the tested isolates

MLST typing revealed that both *E. coli* WZ33 and WZ51 belonged to ST167, indicating that they were clonally related. *E. coli* isolates belonging to ST167 have not been reported before in China. To our best of knowledge, this is the first report of *E. coli* ST167 clone in China and NDM-1 in *E. coli* ST167 clone. These two genetically related isolates carried different plasmids carrying *bla*_NDM-1_, indicating that *E. coli* WZ33 and WZ51 acquired *bla*_NDM-1_-carrying plasmids by different ways. ST167 was found to be prevalent ST among ESBL-producing *E. coli* isolates from animals [36,37]. This clone is rare in clinical isolates. We did not know whether *E. coli* WZ33 and WZ51 were from animals and further investigation should be executed. Recently, a novel NDM carbapenemase variant, NDM-7, was identified in a *E. coli* clinical isolate belonging to ST167 in France [38]. Therefore, emergence of NDM-producing *E. coli* ST167 isolates should be of concern.

## Conclusions

In conclusion, the present study is the first report of *bla*_NDM-1_ carriage in *E. coli* ST167 isolates and coexistence of *bla*_NDM-1_ and *bla*_CMY-42_ in same isolate. Systemic surveillance should focus on the dissemination of *bla*_NDM-1_ among Enterobacteriaceae, especially *E. coli* ST167 clone associated with animal infection.

## Competing interest

The authors declare that they have no competing interests.

## Authors’ contributions

XQZ, DPL, YYX, YPS and DL performed the laboratory measurements. FYY and LXW made substantial contributions to conception and design. FYY and LXW revised the manuscript critically for important intellectual content. XYH, YPL and LHH participated in design and coordination. FYY drafted the manuscript. All authors read and approved the final manuscript.
